# A Revised Classification of Vesicular Stomatitis Virus (VSV) Genotypes and Subtypes

**DOI:** 10.3390/pathogens15070689

**Published:** 2026-06-30

**Authors:** Bernal León, Gabriel González, Bradd Mendoza-Guido, Consuelo Carrillo, Luis L. Rodríguez, Kathryn A. Hanley, Nidia S. Trovao

**Affiliations:** 1 Laboratorio Bioseguridad, Departamento Diagnóstico Veterinario, Laboratorio Nacional de Servicios Veterinarios, Servicio Nacional de Salud Animal, Ministerio de Agricultura y Ganadería, Ulloa, Heredia 40104, Costa Rica; 2 Institute for Vaccine Research and Development, Hokkaido University, Sapporo 001-0021, Japan; 3 Instituto de Investigaciones en Salud, Universidad de Costa Rica, Montes de Oca, San José 11501, Costa Rica; bradd.mendoza@ucr.ac.cr; 4 Independent Researcher, Westbrook, CT 06498, USA; consca70@gmail.com; 5 Department of Biology, New Mexico State University, Las Cruces, NM 88003, USA; luisvsv@gmail.com (L.L.R.); khanley@nmsu.edu (K.A.H.); 6 Department of Pathobiology, College of Veterinary Medicine, University of Illinois Urbana-Champaign, Urbana, IL 61802, USA; 7 Carl R. Woese Institute for Genomic Biology, University of Illinois Urbana-Champaign, Urbana, IL 61802, USA; 8 National Center for Supercomputing Applications, University of Illinois Urbana-Champaign, Urbana, IL 61802, USA

**Keywords:** vesicular stomatitis virus, Indiana virus, New Jersey virus, phylogenetic analysis, viral taxonomy

## Abstract

Vesicular stomatitis virus (VSV) causes clinical disease in livestock that mimics Foot-and-Mouth Disease, necessitating its status as a reportable pathogen to the World Organization for Animal Health. Given the importance of accurate classification for epidemiological surveillance, this study aims to update VSV taxonomic organization using whole-genome sequence criteria to better monitor disease dynamics. Using phylogenetic analysis and the Species Demarcation Tool (SDT), we analyzed pairwise identity across publicly available sequences, constructing a rooted maximum likelihood tree to cluster strains based on robust genetic identity scores. The results demonstrate that nucleotide divergences exceeding 30% define distinct species within the Vesiculovirus genus. Within a species, divergences between 10% and 30% successfully delineate genotypes, while differences between 6% and 10% identify specific subtypes. These quantitative benchmarks provide a precise framework for viral classification, significantly enhancing genomic surveillance and the ability to track the evolution and transmission of VSV genotypes and subtypes across diverse geographic regions.

## 1. Introduction

Vesicular stomatitis viruses (VSV) are arthropod-borne viruses (arboviruses) in the Vesiculovirus genus (family Rhabdoviridae). The VSV genome is characterized by a single non-segmented negative-strand RNA molecule of approximately 11,000 nucleotides in length. It encodes five major structural proteins: nucleocapsid (N), phosphoprotein (P), matrix (M), glycoprotein (G), and the large RNA-dependent RNA polymerase (L), as well as two non-structural proteins of undetermined function, C and C′, encoded from overlapping reading frames in the P gene [1].

VSV sequences show significant genetic variability across the genome [2]. The viral RNA-dependent RNA polymerase lacks proofreading activity; consequently, VSV accumulates one mutation per genome per generation on average, and this diversity may facilitate adaptation to new hosts [3].

The International Committee on Viral Taxonomy (ICTV) utilizes the following criteria to demarcate VSV species (Genus: Vesiculovirus | ICTV): (i) amino acid sequence divergence ≥ 20% in the L protein; (ii) amino acid sequence divergence ≥ 10% in the N protein; (iii) amino acid sequence divergence ≥ 15% in the G protein; (iv) distinguishable via serological tests; and (v) differ in vertebrate hosts and or arthropod vectors. Of the approximately 22 VSV species identified to date, Vesiculovirus stomatitis Indiana (IN) virus (VSIV) and Vesiculovirus stomatitis New Jersey (NJ) virus (VSNJV) [4], both endemic to the Americas, are the major etiological agents of livestock disease and, consequently, the most frequently isolated and sequenced [5].

VSNJV has been previously categorized into six groups (1 to 6) based on a phylogenetic tree topology inferred from phosphoprotein gene sequences [6]. VSIV was initially classified into three groups or clusters according to geographical location: North America, Central America, and South America [7]. Additionally, Bilsel and Nichol classified the Indiana species into four subtypes based on glycoprotein gene comparisons [8]. Subtype 1 comprises strains from the USA, Mexico, and Honduras. Subtype 2 includes two strains from Honduras and Panama without complete genome sequences in GenBank. Subtype 3 includes strain 84-CR-B from Costa Rica isolated in 1984, which shares 99.4% nucleotide identity with genomes MH919396 and MH919397, also from Costa Rica and isolated in 1987. Subtype 4 contains only one strain, 59-PN-L, isolated in Panama in 1959; its closest available genome sequence, with 81.6% identity, is MH919396 isolated from Costa Rica in 1987. However, this reliance on different genetic markers and criteria for each species has created phylogenetic ambiguities that can now be resolved with a standardized, genomics-based approach.

Recent advances and cost reductions for sequencing technologies have made whole-genome sequence characterization of VSVs more feasible [9]. Whole-genome analysis can expose inconsistencies in traditional taxonomic classifications that rely on more limited data. Based on complete-genome nucleotide divergence and phylogenetic reconstruction [10], we propose a revised classification of genotypes and subtypes for both Vesicular stomatitis New Jersey virus (VSNJV) and Vesicular stomatitis Indiana virus (VSIV).

## 2. Materials and Methods

### Pairwise Sequence Identity and Phylogenetic Clustering

For this study, all previously described genotypes, subtypes, and groups of the VSIV and VSNJV species were used as reference sequences to perform BLAST v2.17.0 searches against all complete genome sequences available in the GenBank, European Nucleotide Archive (ENA), and the DataBank of Japan (DDBJ) databases [11]. In the case of VSNJV, we based our classification on a previous phylogenetic study using the phosphoprotein gene, which divided this virus into six groups designated by the Roman numerals I–VI, with Group I further subdivided into subgroups Ia and Ib [6]. In contrast, glycoprotein sequences of VSINV were classified as subtypes into four groups, designated I–IV [12].

As of July 2025, a total of 1070 VSV sequences had been deposited in these databases, of which 215 belonged to the VSNJ and VSI viruses. A total of 117 whole-genome sequences, representing 54.4% of the available NJ and IN genome sequences, were downloaded for analysis. Supplementary Table S1 provides accession numbers for these sequences.

Of the 117 genomes analyzed, two of the three Costa Rican sequences were obtained through VSV surveillance conducted between 2008 and 2024 (unpublished data), while the third was isolated in 2009 from the brain of a cow with encephalitis as part of a surveillance program for encephalitis in cattle and horses carried out between 2009 and 2019 [13].

In addition to the 117 whole-genome sequences, three Vesiculovirus sequences were included to root the phylogenetic tree: HM627187 (Chandipura virus), NC_028255 (Cocal virus), and EU373658 (Alagoas virus). All 120 sequences were aligned using the Clustal Omega tool (https://www.ebi.ac.uk/jdispatcher/msa/clustalo, accessed on 23 June 2026) [14]. Editing of the 120 genomes was performed as follows: the non-coding regions at the 5′ and 3′ ends were removed, and the phosphoprotein gene sequences were extracted for a separate analysis using BioEdit v 7.1.1 [15].

Potential recombination events were assessed using RDP4 v4.97. A sequence was considered recombinant if any recombination event was significantly supported (*p* < 0.05) by at least four of the seven algorithms implemented in RDP4. In cases of suspected recombination, the sequence was removed from subsequent analyses [16]. Pairwise sequence identities and standard deviation for each group of genome sequences were estimated using the Species Demarcation Tool (SDT) v1.3, which performs pairwise alignments with MUSCLE, calculates pairwise identity scores by determining the proportion of mismatched nucleotides across aligned sequences, excluding gaps [17].

A rooted maximum likelihood phylogenetic tree was constructed to cluster closely related sequences based on identity scores. To assess differences in tree topology and identity threshold values between the previous and proposed genotype classifications, we compared phylogenetic trees inferred for the whole-genome sequences and phosphoprotein gene sequences. The best-fit substitution models, as determined using the MEGA 12 [18], were GTR+G4+I for the whole-genome sequences dataset and HKY+G for the phosphoprotein gene. These models were applied to construct maximum likelihood phylogenetic trees with 1000 bootstrap replicates using MEGA 12 [18].

To further assess the robustness of the proposed VSV classification, 307 complete or partial phosphoprotein sequences (36% of 856 available sequences) Table S2 were retrieved from the National Center for Biotechnology Information (NCBI) database based on BLASTn v2.17.0 results obtained by searching each genome representing the genotypes proposed in this study. In total, 307 phosphoprotein sequences were aligned using Clustal Omega [14].

Furthermore, additional phylogenetic analyses were performed to compare tree topologies obtained using different methods. The first analysis was conducted with IQ-TREE v3.0.1 [19], which implements the maximum likelihood method. The GTR+G4+I model was selected as the best-fit nucleotide substitution model, and branch support was assessed using 1000 bootstrap replicates. The resulting tree was evaluated with TempEst v1.5.3, visualized in FigTree v1.4.4, exported in Newick format, and subsequently edited in MEGA 12.

A second analysis was performed using BEAST v1.10.4 [20], which employs Bayesian inference. The analysis used the GTR+G+I substitution model, a strict molecular clock, and a coalescent constant-size tree prior. Markov chain Monte Carlo (MCMC) chains were run for 100 million generations. The resulting Bayesian phylogeny was then compared with the maximum likelihood trees generated by MEGA 12 [18] and IQ-TREE to assess the consistency of tree topology and clade support.

## 3. Results

Although sequence ON805824-026_NJ_2010 did not strictly satisfy the predefined criteria for classification as a recombinant, as only three of the seven algorithms identified it as such; therefore, it was excluded from the whole-genome analysis out of caution. Moreover, the intrinsic biological properties of RNA viruses make the reliable confirmation of true recombination events difficult. No recombination events were detected in the phosphoprotein gene obtained from this genome or among the additional 307 phosphoprotein sequences analyzed.

**Figure 1:**
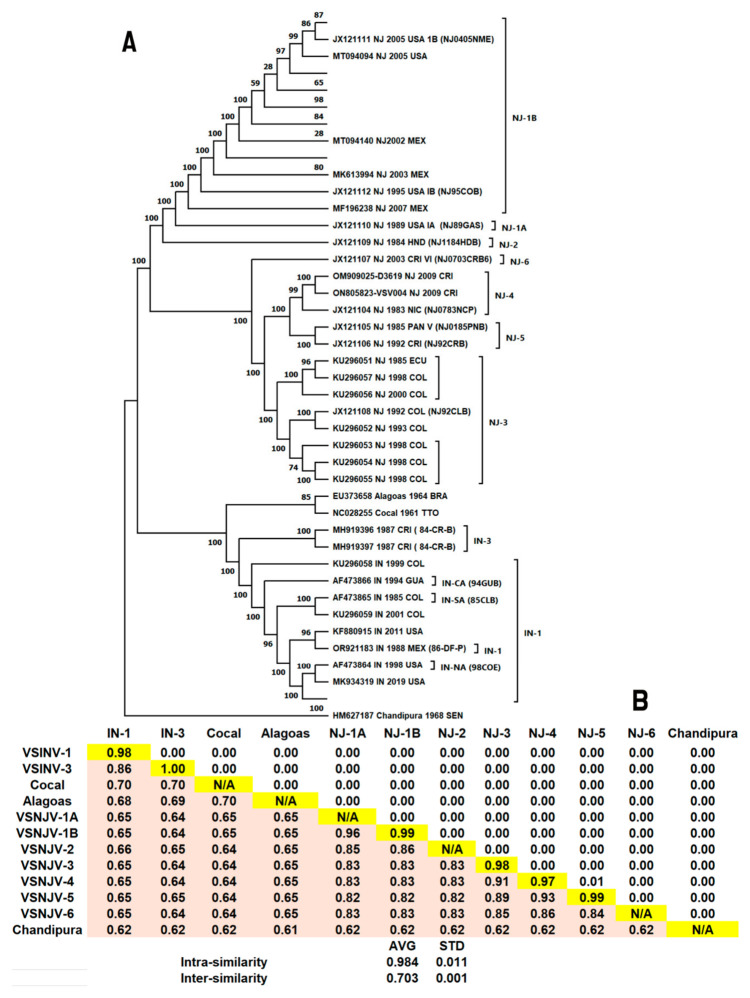
Cladogram tree and pairwise identity matrix of the VSV complete genomes. (**A**) Phylogenetic analysis of 119 complete VSV genomes. Six clusters into the group NJ-1B were condensed to reduce the size of the cladogram. (**B**) Pairwise identity matrix. Values below the diagonal in light pink highlight are the identity scores between groups; within-group estimates are shown along the diagonal in yellow highlight, with “N/A” denoting values that cannot be calculated because only one sequence is present in the group, and standard deviations are displayed above the diagonal.

The VSNJV and VSIV genome sequences were distributed across the same number of clades previously described [6,7,8]. Figure 1A shows the distribution of 119 genome sequences according to the previously defined NJ and IN groups. Reference sequences for each group are indicated in parentheses. To determine the intra- and intergroup identity scores. The NJ viruses were divided into six groups, with NJ-1 further subdivided into two subgroups—NJ-1A and NJ-1B, as they were classified previously [6]—to determine the identity score into and inter these groups. For readability, a cladogram is shown, but the phylogenetic tree with the branch lengths is available as Supplementary Figure S1A.

The IN sequences were classified into four groups based on the glycoprotein gene, as previously described [8]. However, because neither phosphoprotein nor complete genome sequences for the glycoprotein-based subtypes 2 and 4 are available in the GenBank database, only the IN-1 and IN-3 groups are represented in Figure 1A. Interestingly, sequence KU296058, isolated in Colombia, does not cluster with KU296059 and AF473865, which were also isolated in Colombia. Instead, this sequence shares a common ancestor with the other IN-1 sequences, supported by a bootstrap value of 100. Figure 1B presents the corresponding pairwise identity matrix, which quantifies the genetic distances among the established groups.

The smallest interspecies nucleotide divergence (30%) was observed among the Cocal, Indiana, and Alagoas species, confirming that they share a common origin; however, they are clearly distinct species. The highest intragroup variation within the same species (3%) was found in group NJ-4, while the smallest inter-group difference within the same species (7%) was observed between NJ-4 and NJ-5 groups.

**Figure 2:**
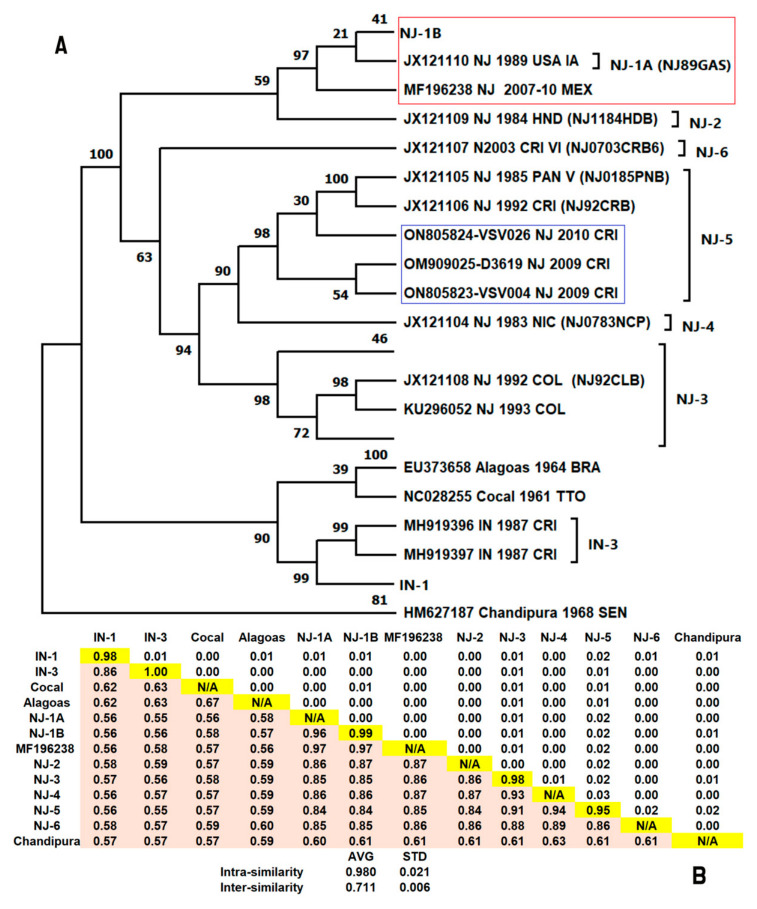
Analysis of 120 phosphoprotein (P) gene sequences derived from the complete genomes shown in Figure 1. Panel (**A**) shows the topology of the VSV sequences distributed by groups. Rectangles highlight the main conflicts between the phosphoprotein gene and whole-genome phylogenies. Panel (**B**) shows the matrix of the pairwise identity scores. Values below the diagonal in light pink highlight are the identity scores between groups; within-group estimates are shown along the diagonal in yellow highlight, with “N/A” denoting values that cannot be calculated because only one sequence is present in the group, and standard deviations are displayed above the diagonal.

Figure 2 shows the groups distribution based on the 120 phosphoprotein (P) gene sequences, some clusters are collapsed.

Two key differences were noted when comparing this topology (Figure 2) to the whole-genome phylogenetic tree (Figure 1). The red frame highlights two sequences, MF196238 and JX121110, which share a common ancestor with the NJ-1B clade (comprising 69 condensed sequences). While MF196238 clustered within the NJ-1B group in the whole-genome topology (Figure 1), it shares a common ancestor with both the NJ-1A and NJ-1B groups in the phosphoprotein gene tree. A second inconsistency, indicated by the blue rectangle, involves several Costa Rican strains, including ON805824 (previously excluded from the genome analysis), ON805825, and ON805826. In the phosphoprotein gene topology, these sequences cluster within NJ-5, whereas the whole-genome topology (Figure 1A) places them within the NJ-4 group. Overall, the genetic distance between the NJ-1A and NJ-1B subgroups was 4% in both the phosphoprotein gene and the complete genome datasets (Figure 1B and Figure 2B). In contrast, the intra-group identity score for NJ-5 was 5% in the phosphoprotein gene tree compared to only 1% in the complete genome analysis (Figure 2B and Figure 1B, respectively). Furthermore, the pairwise identity matrix revealed a divergence of 6% between the NJ-4 and NJ-5 groups in the phosphoprotein gene, while this divergence reached 7% at the whole-genome level.

**Figure 3:**
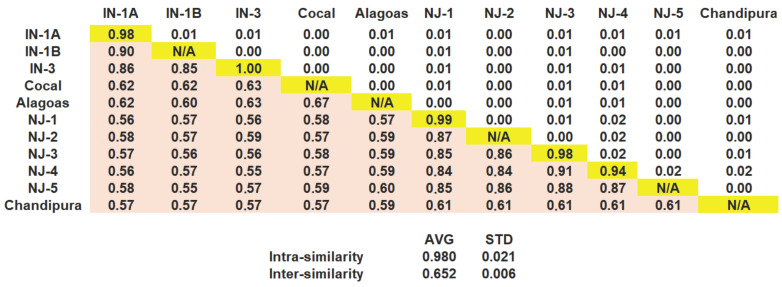
Pairwise identity matrix of 120 phosphoprotein gene sequences under the revised classification system. Values below the diagonal in light pink highlight are the identity scores between groups; within-group estimates are shown along the diagonal in yellow highlight, “N/A” denotes values that cannot be calculated because only one sequence is present in the group, and standard deviations are displayed above the diagonal.

Based on these results and the topology shown in Figure 2A, we propose merging the NJ-4 and NJ-5 groups into a single group, the new NJ-4 group. Additionally, we recommend merging subgroups NJ-1A and NJ-1B into a single group, NJ-1. We also suggest the addition of a new subtype into the Indiana group, designated IN-1B, which includes the sequence KU296058 from Colombia. The impact of these changes is illustrated in a pairwise identity matrix (Figure 3).

The matrix in Figure 3 reflects the proposed taxonomic updates, including the incorporation of subtype IN-1B into genotype IN-1, the consolidation of NJ-1A and NJ-1B into genotype NJ-1, and the merging of NJ-4 and NJ-5 into genotype NJ-4, with NJ-5 replacing the previous designation NJ-6. These revised groupings, hereafter referred to as genotypes, provide a clearer delineation from the previously defined groups.

Under this framework, the minimum intergenotypic nucleotide difference increases to 9% (between NJ-3 and NJ-4), up from the 7% observed prior to the merger of NJ-4 and NJ-5. Furthermore, the intragroup identity score for the newly defined NJ-4 is 6%, representing a 1% increase over the former NJ-5 group alone (Figure 2B). The high intra-group identity scores, such as the 99% observed for the consolidated NJ-1 genotype, further validate these taxonomic modifications.

The highest nucleotide divergence observed within the VSIV groups was 15% between the IN-3 genotype (hereafter renamed IN-2) and the newly proposed IN-1B subtype. In contrast, the divergence between the proposed IN-1B subtype and IN-1A was less than 10% (9.8%). Based on the results obtained from the whole-genome sequences and the 120 phosphoprotein gene sequences, genetic divergences greater than 30% were used to define species. Divergences in identity scores greater than 10% and up to 30% were considered indicative of distinct genotypes, whereas divergences between 6% and 10% were used to define subtypes within a genotype.

**Figure 4:**
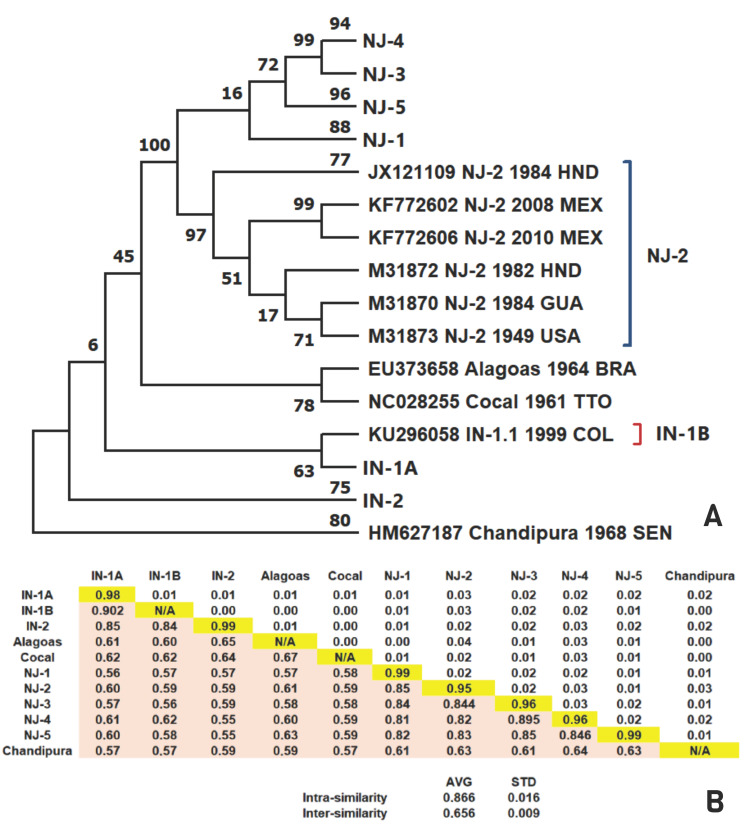
Validation of the revised VSV classification using an expanded dataset of 307 phosphoprotein sequences. (**A**) Phylogenetic tree based on 307 sequences; for clarity, nodes containing more than 10 sequences have been condensed. (**B**) Pairwise identity matrix organized according to the proposed VSV classification for the 307 phosphoprotein sequences. Values below the diagonal in light pink highlight are the identity scores between groups; within-group estimates are shown along the diagonal in yellow highlight.

Figure 4A presents the cladogram constructed from 307 complete and partial phosphoprotein gene sequences, while Figure 4B shows the pairwise identity scores among these sequences according to the proposed species, genotype, and subtype classification criteria.

The phylogenetic tree demonstrates that the proposed genotype and subtype structure remains robust when tested against a larger, more diverse dataset. The IN-1A genotype comprises sequences, including single isolates from Mexico (1998), Guatemala (1994), Ecuador (1994), and Honduras (1983); two each from Colombia (1985–2001) and El Salvador (1971–1985); and 88 sequences isolated in the United States between 1998 and 2020. The IN-1B group (red bracket Figure 4A), consists of a single Colombian sequence (KU296058), which shares 90% identity with its closest relative, KU296059 (also from Colombia, IN-1 2001), according to BLAST analysis [21]. Notably, the IN-2 group contains 15 sequences exclusively from Costa Rica, spanning over three decades (1979–2012). Despite the large sample size and diverse geographic origins, the intragroup identity for IN-1A was 98%, while IN-2 showed a slightly higher identity of 99%, consistent with its localized origin (Figure 4B).

The NJ sequences are distributed across the five proposed groups. The NJ-1 group comprises 77 sequences, primarily from the United States (1952–2012) and 10 from Mexico (2000–2008). The NJ-2 group (blue bracket Figure 4A), expanded from one to six sequences, including the 1984 Honduras reference (JX121109), sequences from Mexico (2008–2010), the USA (1949), Guatemala (1984), and an additional isolate from Honduras (1982). The six sequences belonging to the NJ-2 genotype showed 95% intra-genotype identity. The smallest inter-genotype distance observed among the NJ groups was 10.5%, between NJ-3 and NJ-4, which is consistent with the proposed genotype cutoff.

The NJ-3 group consists of 15 sequences, 12 of which originate from South America (Ecuador and Colombia, 1976–2018), with the remaining three from Central America (1982). The average intragroup identity was 96%. Similarly, the NJ-4 group includes 84 sequences from Panama, Costa Rica, and Nicaragua, maintaining a high intragroup identity of 96% despite the large dataset. Finally, the NJ-5 group is composed of 10 Central American sequences from Honduras and Costa Rica (1986–2009). The minimum difference between NJ-5 and any other NJ group was higher than 14% (observed with both NJ-3 and NJ-4), reinforcing its status as a distinct genotype. The intra-group identity score for NJ-1 was 99%, and the smallest identity difference between NJ-1 and any other group or species was 15% against NJ-2.

Finally, no differences were observed in the topology of the VSV genotypes or subtypes when the phylogenetic trees generated by IQ-TREE and BEAST were compared with the tree constructed using MEGA 12 (Figure S3).

## 4. Discussion

Establishing a viral classification system is essential to provide an organized framework and reference guide for researchers working on vesicular stomatitis virus (VSV). Such a system should include representative reference sequences for the different genotypes and subtypes, enabling researchers to determine which viral variants are circulating in specific geographic regions. Ideally, a classification system should be sufficiently robust to be applied consistently across both individual genes and complete genome sequences.

For this reason, we initially focused on the whole-genome reference sequences previously described in the literature. However, one of the main limitations encountered was the relatively small number of complete genome sequences available in public databases. In contrast, phosphoprotein sequences were considerably more abundant. Therefore, the next logical step was to compare the phylogenetic topology generated from these reference sequences using both whole-genome and phosphoprotein datasets, applying statistically robust phylogenetic approaches based on the maximum likelihood method and the best-fit substitution model.

Once the sequence groups were established, we compared the topologies obtained from the whole-genome and phosphoprotein phylogenetic trees and evaluated the identity scores within and between these groups for both datasets. Based on these analyses, the proposed genotypes and subtypes were defined.

Finally, to validate this classification system, we expanded the analysis by incorporating 307 phosphoprotein sequences, representing a 155% increase in the number of sequences analyzed. This larger dataset confirmed that the proposed genotype and subtype classification, as well as the established cutoff values, were sufficiently robust and versatile to correctly incorporate all available sequences.

The results obtained in this study provide compelling evidence supporting the need to revise the current genotype classification of VSV. Establishing a viral classification system is essential to provide an organized framework and reference guide for researchers working on vesicular stomatitis virus (VSV). Such a system should include representative reference sequences for the different genotypes and subtypes, enabling researchers to determine which viral variants are circulating in specific geographic regions. Ideally, a classification system should be sufficiently robust to be applied consistently across both individual genes and complete genome sequences.

The inconsistencies observed between the two approaches were resolved through the newly proposed classification (Figure S2).

Traditionally, due to its hypervariable domain, the phosphoprotein gene has served as a primary marker for VSV taxonomy and phylogenetic analysis [22,23,24,25]. Therefore, over the years, an abundance of sequences has accumulated for this gene, offering a solid base for the identification of novel genotypes or subtypes.

A crucial result of this research is that the proposed identity thresholds validate the phosphoprotein (P) gene as a reliable tool for VSV surveillance, especially in low- and middle-income countries where resources are limited, especially whole-genome sequencing [24]. By resolving the conflicts between P-gene and whole-genome phylogenies (Figure 1 and Figure 2), this updated system (Figure 3 and Figure 4) allows for the continued use of the hundreds of partial and/or complete P-gene sequences available in public databases, ensuring that historical data remains relevant within a modernized taxonomic framework.

The inclusion of a larger number of sequences, many of which correspond to fragments of the phosphoprotein gene, was crucial for testing the robustness and flexibility of the proposed VSNJV and VSIV classification systems. The observed phylogenetic topology in Figure 4A was supported by the identity scores shown in the corresponding table in Figure 4B.

However, despite the increasing number of available sequences, some subtypes are still represented by a single sequence because no additional related sequences are currently available in the database. The IN-1B subtype is represented by sequence KU296058. According to GenBank, the closest related sequence is KU296059, also isolated in Colombia, sharing 90.26% nucleotide identity. The second most similar sequence is AF473865, likewise isolated in Colombia, with 90.11% identity. Consequently, both sequences were classified within subtype IN-1A. These sequences and their respective subtypes share a common ancestor with a bootstrap support value of 63 (Figure 4A).

The smallest genetic distance observed between distinct species was 33%, found between the Alagoas and Cocal viruses. The smallest distance between VSNJV and VSIV species was 38%, observed between NJ-4 and IN-2. Based on these findings, we propose that nucleotide differences equal or greater than 30% should be used to define separate species within the Vesiculovirus genus.

In contrast, the smallest genetic distance observed between groups within the same genotype was 9.8%, detected between VSIV-1A and VSIV-1B. The smallest distance observed between genotypes was 10.5% between VSNJV-3 and VSNJV-4. Accordingly, under the newly suggested VSV framework, nucleotide differences of 10% to 30% serve as the appropriate thresholds for defining distinct genotypes within a single species. Figure S2 shows the identity scores of the genomes distributed according to the proposed classification.

Finally, the smallest distance between subgroups within a single genotype was 5%, which differs from the 4% initially proposed (Figure 4A,B) observed between NJ-2A and NJ-2B. Therefore, we propose that nucleotide differences greater than 5% but less than 10% should be the criterion for identifying subtypes within a genotype.

Inconsistencies between the phosphoprotein gene tree and the complete genome tree suggest that classifications based solely on phylogenetic topology may be inaccurate. Alternatively, classification approaches that combine phylogenetic tree topology with analyses of genetic divergence within and between groups, particularly when applied to complete genomes, have been successfully implemented in the taxonomy of several viruses. The proposed classification was consistently supported regardless of the phylogenetic software or inference method employed, confirming the robustness of the results.

For example, hepatitis B virus (HBV) genotypes are classified according to the percentage of nucleotide sequence divergence across the complete viral genome. The current molecular classification system recognizes 10 distinct genotypes (A–J) and more than 40 subtypes. Genotypes are defined by an intergroup nucleotide divergence greater than 7.5–8% across the complete genome, whereas subtypes are defined by an intra-genotype divergence ranging from 4% to 7.5%. Importantly, this classification system has remained stable over time, demonstrating the robustness of combining phylogenetic and divergence-based criteria for viral classification [26,27,28,29,30].

A striking finding of this study is the high intragroup identity (98–99%) within the IN-1A subtype and NJ-1 genotypes, despite these sequences being collected over several decades. These genotypes are primarily responsible for outbreaks in the United States and Mexico. The high level of conservation suggests a "stasis-and-outbreak" cycle, where the virus likely persists in stable ecological niches or insect vectors in endemic regions of Mexico before periodically migrating northward. This contrasts with the higher diversity seen in Central and South American genotypes (NJ-3 and NJ-5), where multiple distinct lineages appear to co-circulate within smaller geographic footprints like Costa Rica and Colombia.

The decision to consolidate NJ-1A/NJ-1B and NJ-4/NJ-5 into single genotypes was driven by our proposed 10% threshold. Before consolidation, the intergenotypic difference between NJ-4 and NJ-5 was 9% in the whole genomes (Figure 1B) and 7% in the phosphoprotein gene (Figure 2B), which fall below the threshold for separate genotypes but fits perfectly within our definition of a subtype. The identification of IN-1B as a new subtype containing Colombian isolates further emphasizes that South American VSV diversity remains under-sampled and complex.

Before conducting phylogenetic analyses, it is important to identify potential recombinant sequences because recombination can alter gene and genome structure, thereby affecting the inferred evolutionary relationships among sequences. Although homologous recombination has occasionally been reported in negative-sense RNA viruses, it is generally considered rare, and many reported events may result from laboratory contamination or bioinformatic artifacts rather than true biological recombination. Therefore, recombination signals in these viruses should be interpreted cautiously and only after stringent quality control [31]. To minimize false-positive detections, recombination analyses were performed using RDP4, a widely used tool that integrates seven independent algorithms. A recombination event was considered credible only when supported by at least four of the seven methods.

The sequence ON805824 was collected in Costa Rica in 2010 and was isolated and sequenced in Plum Island laboratory during a standardization and validation of a real-time PCR. In the whole genome tree this sequence shares a common ancestor with another sequence ON805825 isolated from a bovine brain in 2009. While in the phosphoprotein gene tree ON805824 share a common ancestor with two sequences one from Costa Rica JX121106 and another from Panama JX121105 collected in 1992 and 1985 respectively. According to three algorithms of RDP4, the major parent was the sequence ON805825 and the minor parent was JX121104 (Figure S4A).

It is important to note that, although this sequence was not included in the whole-genome phylogenetic analysis, Figure S4B (whole-genome) and Figure S4C (phosphoprotein gene) also demonstrate that ON805824 consistently clustered with OM909025, ON805823, JX121105, and JX121106 within the proposed NJ-4 genotype, indicating that its inclusion did not alter the phylogenetic classification and further confirming the robustness of the proposed classification.

## 5. Conclusions

This study establishes an updated taxonomic classification for VSV based on robust genomic criteria derived from both whole-genome and phosphoprotein gene sequences. Moreover, phylogenetic analyses performed using different approaches, including Bayesian inference, produced consistent topologies for the proposed VSV genotypes and subtypes, supporting the reliability of the classification framework. The aim of this system is to improve the categorization of VSV strains and provide a standardized tool for epidemiological, clinical, and virological research.

Ultimately, the proposed classification scheme will facilitate the tracking of VSV outbreaks, strengthen molecular epidemiological investigations, and contribute to more effective surveillance and control strategies.

## Data Availability

The original contributions presented in this study are included in the article/Supplementary Materials. Further inquiries can be directed to the corresponding authors.

## Supplementary Materials

The following supporting information can be downloaded at: https://www.mdpi.com/article/10.3390/pathogens15070689/s1, Figure S1. Phylogenetic trees with branch lengths for key datasets. Figure S2. Shows the phylogenetic tree and the identity scores of the 119 whole genome. Figure S3. Comparison of the proposed VSV classification phylogenetic trees topologies with branch lengths performed with three different programs. Figure S4. The results obtained using the RDP4 program algorithms, which identified sequence ON805824 as a recombinant and the topology of the whole genome and the Phosphoprotein gene. Table S1. Metadata for the 120 complete Vesicular Stomatitis Virus genomes used in this study. Table S2. Metadata for the 307 phosphoprotein Vesicular Stomatitis Virus gene used in this study. All sequences were downloaded from the International Nucleotide Sequence Database Collaboration (GenBank, ENA, DDBJ). Some of the GenBank accession numbers include a reference ID and the corresponding group, as previously described in the literature [6,8]. The following column lists the proposed virus classification. The final two columns show the year of collection and the country of origin.

## Abbreviations

The following abbreviations are used in this manuscript:

VSV	Vesicular Stomatitis Virus
SDT	Species Demarcation Tool
RNA	Ribonucleic acid
ICTV	International Committee on Viral Taxonomy
VSIV	Vesiculovirus stomatitis Indiana virus
VSNJV	Vesiculovirus stomatitis New Jersey virus
