# Supplementary material for: A Revised Classification of Vesicular Stomatitis Virus (VSV) Genotypes and Subtypes
Source: Pathogens. 2026 Jun 30;15(7):689. doi: 10.3390/pathogens15070689 (PMC13414772; doi:10.3390/pathogens15070689)
Supplement: Supplementary file 1 [file pathogens-15-00689-s001.zip › pathogens-4316086-supplementary.pdf]

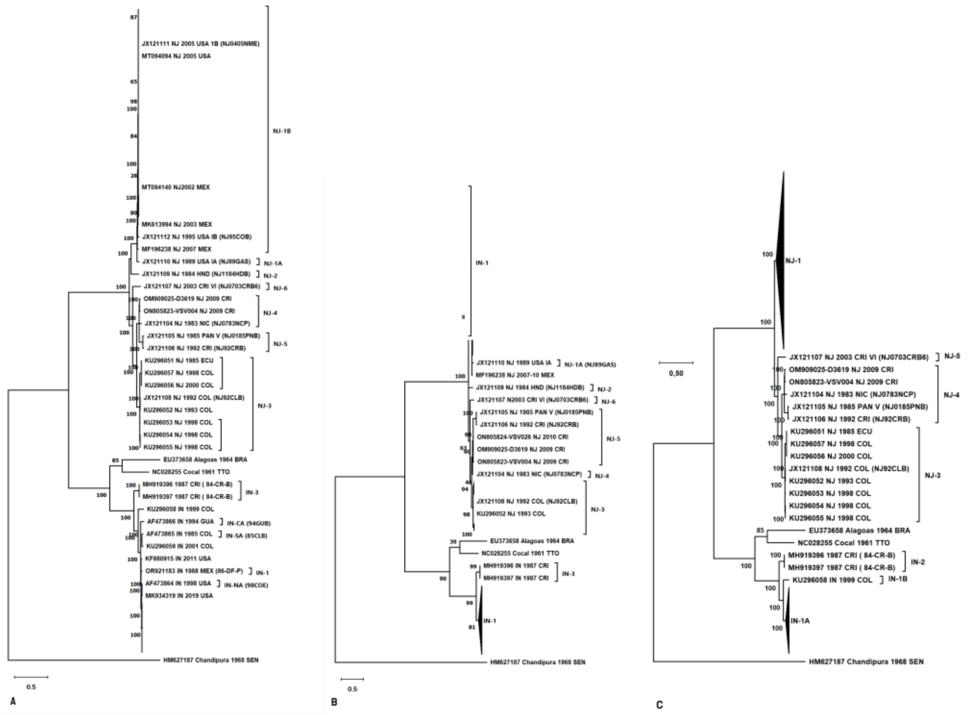

Figure S1. Phylogenetic trees with branch lengths for key datasets. This figure provides full phylogenetic trees, including branch lengths indicating genetic distance, which were condensed for clarity in the main text. (A) Tree of 119 complete VSV genomes under the previous classification. (B) Tree of 120 phosphoprotein gene sequences under the previous classification. (C) Tree of the expanded 307 phosphoprotein gene sequences dataset organized according to the newly proposed classification.

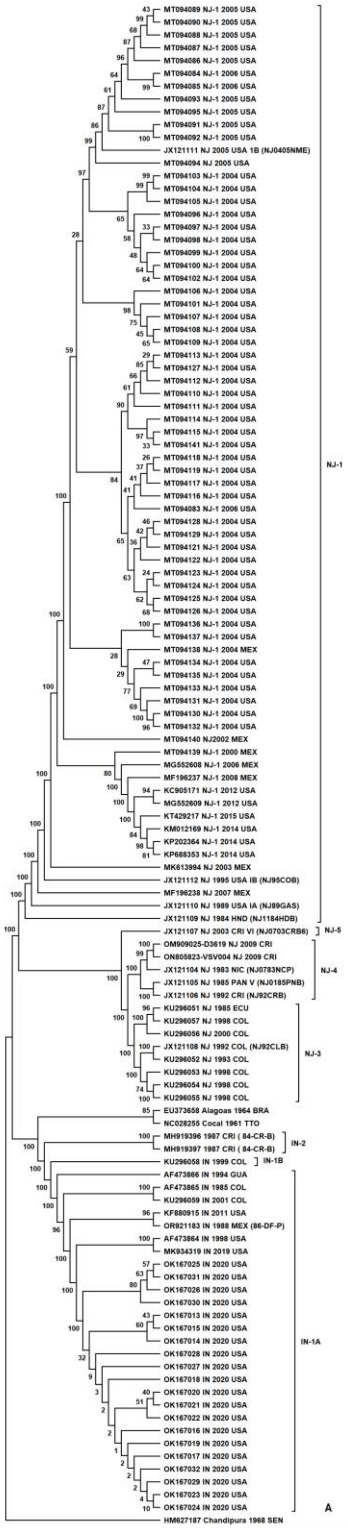

	IN-1A	IN-1B	IN-2	Cocal	Alagoas	NJ-1	NJ-2	NJ-3	NJ-4	NJ-5	Chandipura
IN-1A	0,98	0,00	0,00	0,00	0,00	0,00	0,00	0,00	0,00	0,00	0,00
IN-1B	0,90	N/A	0,00	0,00	0,00	0,00	0,00	0,00	0,00	0,00	0,00
IN-2	0,86	0,85	1,00	0,00	0,00	0,00	0,00	0,00	0,00	0,00	0,00
Cocal	0,70	0,69	0,70	N/A	0,00	0,00	0,00	0,00	0,00	0,00	0,00
Alagoas	0,68	0,68	0,69	0,70	N/A	0,00	0,00	0,00	0,00	0,00	0,00
NJ-1	0,65	0,65	0,64	0,65	0,65	0,99	0,00	0,00	0,01	0,00	0,00
NJ-2	0,66	0,66	0,65	0,64	0,65	0,86	N/A	0,00	0,01	0,00	0,00
NJ-3	0,65	0,65	0,64	0,64	0,65	0,83	0,83	0,98	0,01	0,00	0,00
NJ-4	0,65	0,65	0,64	0,64	0,65	0,83	0,83	0,90	0,95	0,01	0,00
NJ-5	0,65	0,65	0,64	0,64	0,65	0,83	0,83	0,85	0,85	N/A	0,00
Chandipura	0,62	0,62	0,62	0,62	0,61	0,62	0,62	0,62	0,62	0,62	N/A

	AVG	STD
Intra-similarity	0,981	0,020
Inter-similarity	0,695	0,003

B

Figure S2. Shows the phylogenetic tree and the identity scores of the 119 whole genome sequences. Figure S2(A) Topology tree according to the proposed classification. Figure S2 (B) Depicts the matrix with the identity scores of the 119 whole genomes.

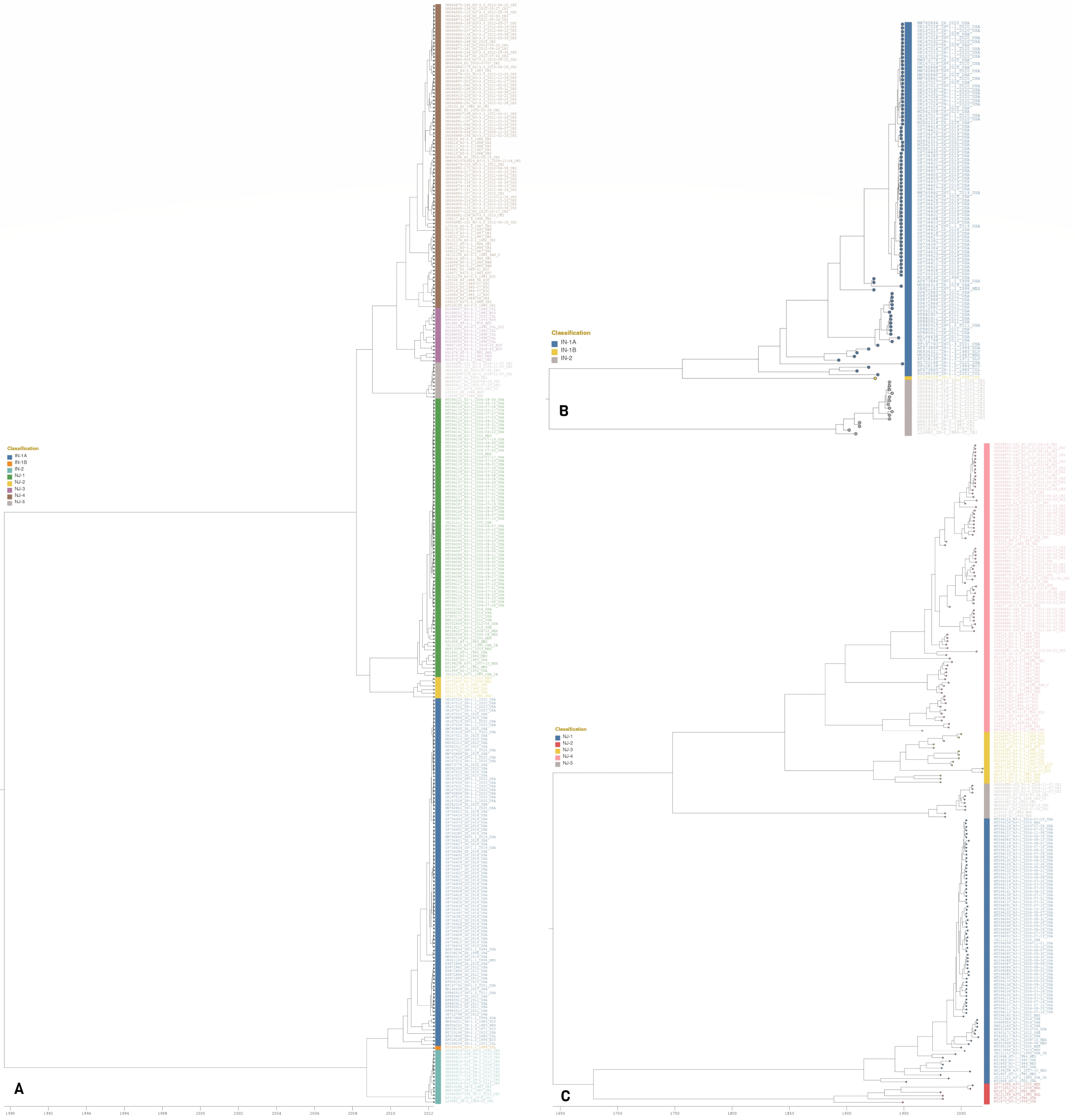

Figure S3. Comparison of phylogenetic tree topologies generated via Bayesian and maximum likelihood inference methods. (A) Bayesian maximum clade credibility tree of 307 phosphoprotein gene sequences inferred using BEAST v.1.10.4, implementing a Yule speciation process as the tree prior, and annotated according to the newly proposed VSV classification system. (B) The corresponding Bayesian phylogenetic tree annotated according to the previous classification framework. (C) The corresponding maximum likelihood phylogenetic tree reconstructed from the same sequence dataset using MEGA 12.

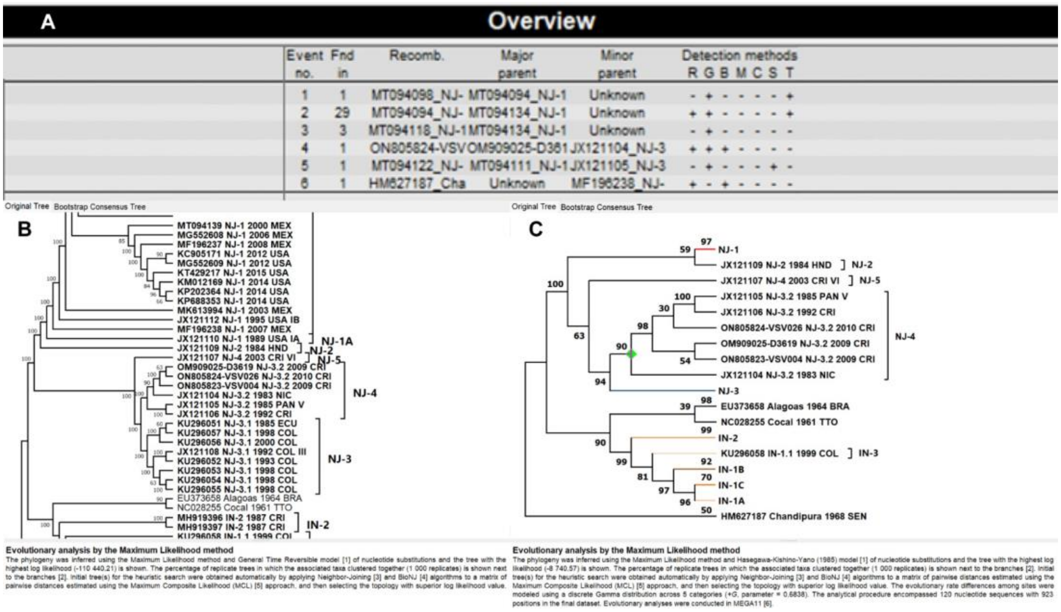

Figure S4. shows the results obtained using the RDP4 program algorithms, which identified sequence ON805824 as a recombinant and the topology of the whole genome and the Phosphoprotein gene. Figure S4A shows the results from the seven algorithms used by RDP4 to classify recombination, R (RDP), G (GENECONV), B (BootScan), M (MaxChi), C (Chimera), S (SiScan) and T (3Seq), recombination are indicated with a + symbol. Figure S4B shows the topology of the complete genome sequence of ON805824 within genotype NJ-4, whereas Figure S4C presents the topology of the phosphoprotein sequence of ON805824 within the same genotype. The text below the cladograms indicates the phylogenetic methods implemented in MEGA 12.

Table S1. Metadata for the 120 complete Vesicular Stomatitis Virus genomes used in this study. All sequences were downloaded from the International Nucleotide Sequence Database Collaboration (GenBank, ENA, DDBJ). Some of the GenBank accession numbers include a reference ID and the corresponding group, as previously described in the literature [6,8]. The following column lists the virus species, and for some entries, the proposed genotype is also indicated. The final two columns show the year of collection and the country of origin.

GenBank ID	Virus Species	Collection year	Country
AF473864- 98COE	VSIV	1998	USA
AF473865-85CLB	VSIV	1985	COL
AF473866-94GUB	VSIV	1994	GUA
EU373658	Alagoas	1964	BRA
HM627187	Chandipura	1968	SEN
JX121104 NJ-4	VSNJV NJ-4	1983	NIC
JX121105 NJ-5	VSNJV NJ-4	1985	PAN
JX121106 NJ-5	VSNJV NJ-4	1992	CRI
JX121107 NJ-7	VSNJV NJ-5	2003	CRI
JX121108 NJ-3	VSNJV NJ-3	1992	COL
JX121109 NJ-2	VSNJV NJ-2	1984	HND
JX121110 NJ1A	VSNJV NJ-1	1989	USA
JX121111 NJ1B	VSNJV NJ-1	2005	USA
JX121112 NJ1B	VSNJV NJ-1	1995	USA
KC905171	VSNJV	2012	USA
KF880915	VSIV	2011	USA
KM012169	VSNJV	2014	USA
KP202364	VSNJV	2014	USA
KP688353	VSNJV	2014	USA
KT429217	VSNJV	2015	USA
KU296051	VSNJV	1985	ECU
KU296052	VSNJV	1993	COL
KU296053	VSNJV	1998	COL
KU296054	VSNJV	1998	COL
KU296055	VSNJV	1998	COL
KU296056	VSNJV	2000	COL
KU296057	VSNJV	1998	COL
KU296058	VSIV IN-2	1999	COL
KU296059	VSIV	2001	COL
MF196237	VSNJV	2008	MEX
MF196238	VSNJV	2007	MEX
MG552608	VSNJV	2006	MEX
MG552609	VSNJV	2012	USA
MH919396 IN-3	VSIV IN-3	1987	CRI
MH919397 IN-3	VSIV IN-3	1987	CRI
MK613994	VSNJV	2003	MEX
MK934319	VSIV	2019	USA

MT094083	VSNJV	2006	USA
MT094084	VSNJV	2006	USA
MT094085	VSNJV	2006	USA
MT094086	VSNJV	2005	USA
MT094087	VSNJV	2005	USA
MT094088	VSNJV	2005	sUSA
MT094089	VSNJV	2005	USA
MT094090	VSNJV	2005	USA
MT094091	VSNJV	2005	USA
MT094092	VSNJV	2005	USA
MT094093	VSNJV	2005	USA
MT094094	VSNJV	2005	USA
MT094095	VSNJV	2005	USA
MT094096	VSNJV	2004	USA
MT094097	VSNJV	2004	USA
MT094098	VSNJV	2004	USA
MT094099	VSNJV	2004	USA
MT094100	VSNJV	2004	USA
MT094101	VSNJV	2004	USA
MT094102	VSNJV	2004	USA
MT094103	VSNJV	2004	USA
MT094104	VSNJV	2004	USA
MT094105	VSNJV	2004	USA
MT094106	VSNJV	2004	USA
MT094107	VSNJV	2004	USA
MT094108	VSNJV	2004	USA
MT094109	VSNJV	2004	USA
MT094110	VSNJV	2004	USA
MT094111	VSNJV	2004	USA
MT094112	VSNJV	2004	USA
MT094113	VSNJV	2004	USA
MT094114	VSNJV	2004	USA
MT094115	VSNJV	2004	USA
MT094116	VSNJV	2004	USA
MT094117	VSNJV	2004	USA
MT094118	VSNJV	2004	USA
MT094119	VSNJV	2004	USA
MT094121	VSNJV	2004	USA
MT094122	VSNJV	2004	USA
MT094123	VSNJV	2004	USA
MT094124	VSNJV	2004	USA
MT094125	VSNJV	2004	USA
MT094126	VSNJV	2004	USA
MT094127	VSNJV	2004	USA
MT094128	VSNJV	2004	USA
MT094129	VSNJV	2004	USA
MT094130	VSNJV	2004	USA
MT094131	VSNJV	2004	USA
MT094132	VSNJV	2004	USA

MT094133	VSNJV	2004	USA
MT094134	VSNJV	2004	USA
MT094135	VSNJV	2004	USA
MT094136	VSNJV	2004	USA
MT094137	VSNJV	2004	USA
MT094138	VSNJV	2004	MEX
MT094139	VSNJV	2000	MEX
MT094140	VSNJV	2002	MEX
MT094141	VSNJV	2004	USA
NC028255	Cocal	1961	TTO
OK167013	VSIV	2020	USA
OK167014	VSIV	2020	USA
OK167015	VSIV	2020	USA
OK167016	VSIV	2020	USA
OK167017	VSIV	2020	USA
OK167018	VSIV	2020	USA
OK167019	VSIV	2020	USA
OK167020	VSIV	2020	USA
OK167021	VSIV	2020	USA
OK167022	VSIV	2020	USA
OK167023	VSIV	2020	USA
OK167024	VSIV	2020	USA
OK167025	VSIV	2020	USA
OK167026	VSIV	2020	USA
OK167027	VSIV	2020	USA
OK167028	VSIV	2020	USA
OK167029	VSIV	2020	USA
OK167030	VSIV	2020	USA
OK167031	VSIV	2020	USA
OK167032	VSIV	2020	USA
OM909025-D3619	VSIV	2009	CRI
ON805823-VSV004	VSIV	2009	CRI
ON805824-VSV026	VSIV	2010	CRI
OR921183 IN-1	VSIV IN-1	1988	MEX

Table S2. Metadata for the 307 phosphoprotein Vesicular Stomatitis Virus gene used in this study. All sequences were downloaded from the International Nucleotide Sequence Database Collaboration (GenBank, ENA, DDBJ). Some of the GenBank accession numbers include a reference ID and the corresponding group, as previously described in the literature [6,8]. The following column lists the proposed virus classification. The final two columns show the year of collection and the country of origin.

GenBank ID	Virus Classification	Collection time	Country
EU373658	Alagoas	1964	BRA
HM627187	Chandipura	1968	SEN
NC028255	Cocal	1961	TTO
AF473864	IN-1A	1998	USA
AF473865	IN-1A	1985	COL
AF473866	IN-1A	1994	GUA
EF028135	IN-1A	1971	SLV
EF028137	IN-2	1979	CRI
EF028138	IN-1A	1994	ECU
EF028139	NJ-4	1966	CRI
EF028147	NJ-3	1976	ECU
EF197793	IN-1A	2001	USA
JX121104	NJ-4	1983	NIC
JX121105-V	NJ-4	1985	PAN
JX121106	NJ-4	1992	CRI
JX121107-VI	NJ-5	2003	CRI
JX121108	NJ-3	1992	COL
JX121109	NJ-2	1984	HND
JX121110-IA	NJ-1	1989	USA
JX121111	NJ-1	2005	USA
JX121112-IB	NJ-1	1995	USA
KC905171	NJ-1	2012	USA
KF772602	NJ-2	2008	MEX
KF772606	NJ-2	2010	MEX
KF880907	IN-1A	2011	USA
KF880910	IN-1A	2011	USA
KF880911	IN-1A	2011	USA
KF880912	IN-1A	2011	USA
KF880913	IN-1A	2011	USA
KF880915	IN-1A	2011	USA
KF935251	IN-1A	2010	USA
KM012169	NJ-1	2014	USA
KP202364	NJ-1	2014	USA
KP688353	NJ-1	2014	USA
KP872882	IN-1A	2012	USA
KP872884	IN-1A	2012	USA
KP872885	IN-1A	2012	USA
KP872886	IN-1A	2012	USA
KP872888	IN-1A	2012	USA
KT429217	NJ-1	2015	USA
KU296051	NJ-3	1985	ECU
KU296052	NJ-3	1993	COL
KU296053	NJ-3	1998	COL

KU296054	NJ-3	1998	COL
KU296055	NJ-3	1998	COL
KU296056	NJ-3	2000	COL
KU296057	NJ-3	1998	COL
KU296058	IN-1B	1999	COL
KU296059	IN-1A	2001	COL
L24963	NJ-4	1982	NIC
L24964	NJ-4	1983	PAN
L24968	NJ-5	1986	HND
L24971	NJ-4	1983	NIC
L24975	NJ-4	1983	PAN
L24981	NJ-4	1985	NIC
L24982	IN-2	1984	CRI
L25006	NJ-4	1987	CRI
L25010	NJ-4	1985	NIC
L25011	NJ-4	1985	NIC
L25014	NJ-4	1986	NIC
L25019	NJ-5	1986	HND
L25033	NJ-4	1986	CRI
L25036	NJ-4	1986	NIC
M31863	NJ-1	1990	USA
M31864	NJ-1	1983	USA
M31865	NJ-1	1984	MEX
M31867	NJ-1	1982	MEX
M31868	NJ-1	1984	MEX
M31869	NJ-1	1952	USA
M31870	NJ-2	1984	GUA
M31872	NJ-2	1982	HND
M31873	NJ-2	1949	USA
M31874	NJ-3	1982	CRI
M31875	NJ-3	1982	HND
M31876	NJ-3	1982	HND
M31879	NJ-4	1990	PAN
M31880	NJ-3	1976	ECU
MF196237	NJ-1	2008	MEX
MF196238	NJ-1	2007	MEX
MG552608	NJ-1	2006	MEX
MG552609	NJ-1	2012	USA
MH919396	IN-2	1987	CRI
MH919397	IN-2	1987	CRI
MK613994	NJ-1	2003	MEX
MK660380	NJ-5	2003	CRI
MK660396	NJ-4	2003	CRI
MK660404	NJ-4	2003	CRI
MK660408	NJ-5	2003	CRI
MK660411	NJ-5	2003	CRI

MK660437	NJ-5	2003	CRI
MK660445	NJ-4	2003	CRI
MK934319	IN-1A	2019	USA
MK934320	IN-1A	1983	HND
MK934321	IN-1A	1985	SLV
MN164438	IN-1A	2017	USA
MT094083	NJ-1	2006	USA
MT094084	NJ-1	2006	USA
MT094085	NJ-1	2006	USA
MT094086	NJ-1	2005	USA
MT094087	NJ-1	2005	USA
MT094088	NJ-1	2005	USA
MT094089	NJ-1	2005	USA
MT094090	NJ-1	2005	USA
MT094091	NJ-1	2005	USA
MT094092	NJ-1	2005	USA
MT094093	NJ-1	2005	USA
MT094094	NJ-1	2005	USA
MT094095	NJ-1	2005	USA
MT094096	NJ-1	2005	USA
MT094097	NJ-1	19/7/2004	USA
MT094098	NJ-1	27/9/2004	USA
MT094099	NJ-1	1/11/2004	USA
MT094100	NJ-1	21/10/2004	USA
MT094101	NJ-1	20/7/2004	USA
MT094102	NJ-1	10/10/2004	USA
MT094103	NJ-1	7/8/2004	USA
MT094104	NJ-1	7/8/2004	USA
MT094105	NJ-1	7/8/2004	USA
MT094106	NJ-1	27/7/2004	USA
MT094107	NJ-1	26/6/2004	USA
MT094108	NJ-1	16/7/2004	USA
MT094109	NJ-1	20/7/2004	USA
MT094110	NJ-1	16/7/2004	USA
MT094111	NJ-1	25/8/2004	USA
MT094112	NJ-1	14/7/2004	USA
MT094113	NJ-1	21/7/2004	USA
MT094114	NJ-1	3/7/2004	USA
MT094115	NJ-1	3/7/2004	USA
MT094116	NJ-1	29/7/2004	USA
MT094117	NJ-1	8/8/2004	USA
MT094118	NJ-1	8/8/2004	USA
MT094119	NJ-1	1/8/2004	USA
MT094121	NJ-1	8/11/2004	USA
MT094122	NJ-1	4/8/2004	USA
MT094123	NJ-1	28/7/2004	USA

MT094124	NJ-1	24/10/2004	USA
MT094125	NJ-1	11/8/2004	USA
MT094126	NJ-1	28/7/2004	USA
MT094127	NJ-1	13/7/2004	USA
MT094128	NJ-1	16/7/2004	USA
MT094129	NJ-1	1/7/2004	USA
MT094130	NJ-1	23/6/2004	USA
MT094131	NJ-1	21/6/2004	USA
MT094132	NJ-1	21/6/2004	USA
MT094133	NJ-1	25/9/2004	USA
MT094134	NJ-1	13/9/2004	USA
MT094135	NJ-1	17/7/2004	USA
MT094136	NJ-1	21/7/2004	USA
MT094137	NJ-1	21/7/2004	USA
MT094138	NJ-1	2004	MEX
MT094139	NJ-1	2000	MEX
MT094140	NJ-1	2002	MEX
MT094141	NJ-1	23/6/2004	USA
MW373778	IN-1A	2020	USA
MW760860	IN-1A	2019	USA
MW760861	IN-1A	2020	USA
MW760864	IN-1A	2020	USA
MW760865	IN-1A	2020	USA
MW760868	IN-1A	2020	USA
MW760869	IN-1A	2020	USA
MZ562308	IN-1A	2020	USA
MZ562310	IN-1A	2020	USA
MZ562312	IN-1A	2020	USA
MZ562313	IN-1A	2020	USA
MZ562318	IN-1A	2020	USA
MZ703188	IN-1A	2020	USA
NC038236	IN-1A	1998	USA
OK167013	IN-1A	2020	USA
OK167014	IN-1A	2020	USA
OK167015	IN-1A	2020	USA
OK167016	IN-1A	2020	USA
OK167017	IN-1A	2020	USA
OK167018	IN-1A	2020	USA
OK167019	IN-1A	2020	USA
OK167020	IN-1A	2020	USA
OK167021	IN-1A	2020	USA
OK167022	IN-1A	2020	USA
OK167023	IN-1A	2020	USA
OK167024	IN-1A	2020	USA
OK167025	IN-1A	2020	USA
OK167026	IN-1A	2020	USA

OK167027	IN-1A	2020	USA
OK167028	IN-1A	2020	USA
OK167029	IN-1A	2020	USA
OK167030	IN-1A	2020	USA
OK167031	IN-1A	2020	USA
OK167032	IN-1A	2020	USA
OM909025-D3619	NJ-4	4/11/2009	CRI
ON084859-004	NJ-4	22/11/2009	CRI
ON084860-026	NJ-4	22/9/2010	CRI
ON084861-043	NJ-4	1/2/2011	CRI
ON084862-048	NJ-4	10/2/2011	CRI
ON084863-133	NJ-4	5/3/2012	CRI
ON084864-134	NJ-4	6/3/2012	CRI
ON084865-135	NJ-4	8/3/2012	CRI
ON084866-136	NJ-4	8/3/2012	CRI
ON084867-137	NJ-4	19/3/2012	CRI
ON084868-138	NJ-4	27/3/2012	CRI
ON084869-139	NJ-4	27/3/2012	CRI
ON084870-140	NJ-4	12/4/2012	CRI
ON084871-141	NJ-4	16/4/2012	CRI
ON084872-142	NJ-4	15/5/2012	CRI
ON084873-143	NJ-4	24/5/2012	CRI
ON084874-144	NJ-4	13/6/2012	CRI
ON084875-146	NJ-4	26/6/2012	CRI
ON084876-147	NJ-4	3/7/2012	CRI
ON084877-151	NJ-4	24/8/2012	CRI
ON084878-152	NJ-4	3/12/2012	CRI
ON084879-153	NJ-4	2012	CRI
ON084880-157	NJ-4	2013	CRI
ON084881-158	NJ-4	2013	CRI
ON084882-161	NJ-4	15/6/2013	CRI
ON084883-168	NJ-4	2010	CRI
ON084884-173	NJ-4	29/6/2013	CRI
ON084885-175	NJ-4	8/4/2010	CRI
ON084886-191	NJ-4	28/1/2010	CRI
ON084887-193	NJ-4	19/1/2011	CRI
ON084888-195	NJ-4	23/2/2012	CRI
ON084889-194	NJ-4	9/2/2010	CRI
ON084890-196	NJ-4	11/2/2011	CRI
ON084891-197	NJ-4	10/2/2011	CRI
ON084892-198	NJ-4	11/5/2011	CRI
ON084893-199	NJ-4	7/4/2011	CRI
ON084894-200	NJ-4	2/4/2011	CRI
ON084895-201	NJ-5	7/11/2009	CRI
ON084896-202	NJ-5	7/11/2009	CRI

ON084897-203	NJ-4	17/1/2011	CRI
ON084898-205	NJ-5	7/11/2009	CRI
ON084899-206	NJ-4	29/11/2011	CRI
ON084900-207	NJ-4	12/4/2012	CRI
ON084901-208	NJ-4	6/3/2012	CRI
ON084902-210	NJ-4	6/4/2011	CRI
ON084903-211	NJ-4	27/6/2012	CRI
ON084904-201	NJ-4	28/12/2010	CRI
ON084905-213	NJ-4	23/12/2010	CRI
ON084906-214	NJ-4	23/12/2010	CRI
ON084907-215	NJ-4	17/10/2010	CRI
ON084908-216	NJ-4	15/4/2011	CRI
ON084909-217	NJ-4	20/2/2011	CRI
ON084910-218	NJ-4	21/2/2011	CRI
ON084911-001	IN-2	2009	CRI
ON084912-007	IN-2	2010	CRI
ON084913-008	IN-2	2010	CRI
ON084914-012	IN-2	2010	CRI
ON084915-014	IN-2	2010	CRI
ON084916-018	IN-2	2010	CRI
ON084917-019	IN-2	2010	CRI
ON084918-150	IN-2	2012	CRI
ON084919-163	IN-2	2010	CRI
ON084920-185	IN-2	2010	CRI
ON084921-189	IN-2	2012	CRI
ON567116	NJ-3	2018-03	ECU
ON567165	NJ-3	2018-10	ECU
OP734363	IN-1A	2019	USA
OP734368	IN-1A	2019	USA
OP734376	IN-1A	2019	USA
OP734382	IN-1A	2019	USA
OP734384	IN-1A	2019	USA
OP734385	IN-1A	2019	USA
OP734397	IN-1A	2019	USA
OP734401	IN-1A	2019	USA
OP734402	IN-1A	2019	USA
OP734403	IN-1A	2019	USA
OP734405	IN-1A	2019	USA
OP734406	IN-1A	2019	USA
OP734407	IN-1A	2019	USA
OP734408	IN-1A	2019	USA
OP734409	IN-1A	2019	USA
OP734410	IN-1A	2019	USA
OP734411	IN-1A	2019	USA
OP734414	IN-1A	2019	USA
OP734417	IN-1A	2019	USA

OP734418	IN-1A	2019	USA
OP734420	IN-1A	2019	USA
OP734422	IN-1A	2019	USA
OP734423	IN-1A	2019	USA
OP734424	IN-1A	2019	USA
OP734425	IN-1A	2019	USA
OP734426	IN-1A	2019	USA
OP734427	IN-1A	2019	USA
OP734428	IN-1A	2019	USA
OP734429	IN-1A	2019	USA
OP734430	IN-1A	2019	USA
OP734431	IN-1A	2019	USA
OP734432	IN-1A	2019	USA
OP734433	IN-1A	2019	USA
OP734434	IN-1A	2019	USA
OP734435	IN-1A	2019	USA
OP734436	IN-1A	2019	USA
OP734439	IN-1A	2019	USA
OR712768	IN-1A	2012	USA
OR921183	IN-1A	1998	MEX
U39209	NJ-4	1989	CRI
U39210	NJ-4	1989	CRI
U39211	NJ-4	1988	CRI
U39212	NJ-4	1987	CRI
U39213	NJ-4	1988	CRI
U39214	NJ-4	1988	CRI
U39215	NJ-4	1988	CRI
U39217	NJ-4	1990	CRI
U39218	NJ-4	1987	CRI
U39219	NJ-4	1987	CRI
U39220	NJ-4	1987	CRI
U39221	NJ-4	1987	CRI
U39222	NJ-4	1986	CRI
U39224	NJ-4	1988	CRI
U39225	NJ-4	1989	CRI
